# Supplementary material for: Predictors of uncertainty and unwillingness to receive the COVID-19 booster vaccine: An observational study of 22,139 fully vaccinated adults in the UK
Source: Lancet Reg Health Eur. 2022 Feb 3;14:100317. doi: 10.1016/j.lanepe.2022.100317 (PMC8811487; doi:10.1016/j.lanepe.2022.100317)
Supplement: Supplementary file 1 [file mmc1.docx]

**Supplementary Material**

**Table S1. Comparison of excluded and included participants, unweighted**

|  | **Excluded** | | **Included** | |
| --- | --- | --- | --- | --- |
|  | **Mean** | **SD** | **Mean** | **SD** |
| Gender |  |  |  |  |
| Male | 0·23 | 0·42 | 0·26 | 0·44 |
| Female | 0·77 | 0·42 | 0·74 | 0·44 |
| Age |  |  |  |  |
| 60+ | 0·36 | 0·48 | 0·43 | 0·50 |
| 45-59 | 0·35 | 0·48 | 0·34 | 0·47 |
| 30-44 | 0·22 | 0·42 | 0·19 | 0·39 |
| 18-29 | 0·07 | 0·25 | 0·04 | 0·19 |
| Ethnicity |  |  |  |  |
| White | 0·95 | 0·21 | 0·97 | 0·18 |
| Ethnic minority groups | 0·05 | 0·21 | 0·03 | 0·18 |
| Education |  |  |  |  |
| Undergraduate degree or higher | 0·64 | 0·48 | 0·71 | 0·46 |
| A-levels or vocational | 0·19 | 0·39 | 0·16 | 0·37 |
| Up to GCSE | 0·17 | 0·38 | 0·13 | 0·34 |
| Income |  |  |  |  |
| >£90,000 | 0·09 | 0·29 | 0·10 | 0·30 |
| £60,000-89,999 | 0·15 | 0·35 | 0·15 | 0·35 |
| £30,000-59,999 | 0·34 | 0·47 | 0·35 | 0·48 |
| £16,000-29,999 | 0·25 | 0·43 | 0·26 | 0·44 |
| <£16,000 | 0·17 | 0·38 | 0·14 | 0·35 |
| Employment status |  |  |  |  |
| Not employed | 0·41 | 0·49 | 0·42 | 0·49 |
| Employed | 0·59 | 0·49 | 0·58 | 0·49 |
| Living arrangement |  |  |  |  |
| Alone | 0·18 | 0·38 | 0·23 | 0·42 |
| With others, not with children | 0·56 | 0·50 | 0·57 | 0·49 |
| With, including children | 0·26 | 0·44 | 0·20 | 0·40 |
| Area of dwelling |  |  |  |  |
| Urban | 0·74 | 0·44 | 0·75 | 0·43 |
| Rural | 0·26 | 0·44 | 0·25 | 0·43 |
| Smoking status |  |  |  |  |
| Former or non-smoker | 0·90 | 0·30 | 0·93 | 0·25 |
| Current smoker | 0·10 | 0·30 | 0·07 | 0·25 |
| Key worker status |  |  |  |  |
| Not a key worker | 0·76 | 0·43 | 0·80 | 0·40 |
| Key worker | 0·24 | 0·43 | 0·20 | 0·40 |
| Long-term physical health condition |  |  |  |  |
| No long-term physical health condition | 0·57 | 0·50 | 0·57 | 0·50 |
| Long-term physical health condition | 0·43 | 0·50 | 0·43 | 0·50 |
| Long-term mental health condition |  |  |  |  |
| No long-term mental health condition | 0·81 | 0·39 | 0·85 | 0·36 |
| Long-term mental health condition | 0·19 | 0·39 | 0·15 | 0·36 |
| Confidence in government to handle pandemic |  |  |  |  |
| Low confidence in government to handle pandemic | 0·31 | 0·46 | 0·33 | 0·47 |
| Low confidence in government to handle pandemic | 0·69 | 0·46 | 0·67 | 0·47 |
| Confidence in health system to handle pandemic |  |  |  |  |
| High confidence in health system to handle pandemic | 0·52 | 0·50 | 0·62 | 0·49 |
| Low confidence in health system to handle pandemic | 0·48 | 0·50 | 0·38 | 0·49 |
| Knowledge of COVID 19 |  |  |  |  |
| High knowledge of COVID-19 | 0·86 | 0·35 | 0·87 | 0·34 |
| Low knowledge of COVID-19 | 0·14 | 0·35 | 0·13 | 0·34 |
| Compliance across strict restriction periods |  |  |  |  |
| Consistently high compliance with COVID-19 guidelines | 0·75 | 0·44 | 0·72 | 0·45 |
| Consistently low compliance with COVID-19 guidelines | 0·25 | 0·44 | 0·28 | 0·45 |
| Have had COVID-19 |  |  |  |  |
| Have not had COVID-19 | 0·74 | 0·44 | 0·79 | 0·41 |
| Have had COVID-19, suspected by self or doctor | 0·12 | 0·32 | 0·10 | 0·30 |
| Have had COVID-19, confirmed by test | 0·14 | 0·34 | 0·11 | 0·31 |
| Stressed in the past week about catching/becoming ill from COVID-19 |  |  |  |  |
| Stressed | 0·32 | 0·47 | 0·35 | 0·48 |
| Not stressed | 0·68 | 0·47 | 0·65 | 0·48 |
| Initial COVID-19 vaccine intention |  |  |  |  |
| Initially willing to accept or had accepted a COVID-19 vaccine | 0·80 | 0·40 | 0·92 | 0·27 |
| Initially uncertain about accepting a COVID-19 vaccine | 0·08 | 0·26 | 0·05 | 0·22 |
| Initially unwilling to accept a COVID-19 vaccine | 0·12 | 0·33 | 0·03 | 0·16 |
| COVID-19 booster vaccine intentions |  |  |  |  |
| Willing/accepted a COVID-19 booster vaccine | 0·85 | 0·36 | 0·95 | 0·21 |
| Uncertain about a COVID-19 booster vaccine | 0·04 | 0·19 | 0·02 | 0·16 |
| Unwilling to accept COVID-19 booster vaccine | 0·11 | 0·32 | 0·02 | 0·15 |

Note. ‘Very likely’ to accept a COVID-19 booster vaccine was the reference group in the multinomial regression model. Ethnic minority groups refers to Black, Asian and minority ethnicity. GCSE refers to General Certificate of Secondary Education.

**Table S2. Wording of study developed items**

| **Variable** | **Question wording** | **Response options** |
| --- | --- | --- |
| Ethnicity | What is your ethnicity? | 1- Asian/Asian British - Indian, Pakistani, Bangladeshi, other  2- Black/Black British - Caribbean, African, other  3- Mixed race - White and Black/Black British  4- Mixed race – other  5- White - British, Irish, other  6- Chinese/Chinese British  7- Middle Eastern/Middle Eastern British - Arab, Turkish, other  8- Other ethnic group  9- Prefer not to say |
| Intention to receive a COVID-19 booster vaccine | We are interested in your thoughts on the BOOSTER VACCINATION·  How likely do you think you are to get a COVID-19 booster vaccine if/when you are offered one? | 1- Very unlikely  6- very likely  I have already had one/accepted one |
| Have ever had COVID-19 | Overall, do you believe you have ever had Covid-19? | Yes, confirmed by a positive Covid-19 test at the time I felt ill  Yes, confirmed by a positive antibody test since  Yes, suspected by a doctor but not tested  Yes, my own suspicions  No, not that I know of |
| Confidence in government to handle pandemic | To what extent do you have confidence in the UK Government’s ability to handle the Coronavirus situation as it continues to develop? | 1- none at all to  7- lots |
| Confidence in health system to handle pandemic | How much confidence do you have that the UK health service can cope during Covid-19? If you live in a devolved nation, we ask you to focus on the health service within your country (e·g·, NHS Health Scotland / NHS Wales / HSCNI)? | 1- none at all to  7- lots |
| Compliance with COVID-19 guidelines | Are you following the recommendations from authorities to prevent spread of COVID-19? | 1- none at all to  7- very much so |
| Knowledge of COVID-19 | How would you rate your knowledge of COVID-19? | 1- very poor knowledge to  7- very good knowledge |

**Table S3. Dates of strict restrictions in England**

| **Restriction period** | **Dates** |
| --- | --- |
| First | 23 March to 9 May 2020 |
| Second | 2 November to 3 December 2020 |
| Third | 5 January to 12 April 2021 |

**Table S4. Comparison of sample participants by COVID-19 booster vaccine intention (weighted, *N* = 22,139)**

|  | **Willing** | | **Uncertain** | | **Unwilling** | |
| --- | --- | --- | --- | --- | --- | --- |
|  | **Mean** | **SD** | **Mean** | **SD** | **Mean** | **SD** |
| Gender |  |  |  |  |  |  |
| Male | 0·50 | 0·50 | 0·42 | 0·49 | 0·43 | 0·50 |
| Female | 0·50 | 0·50 | 0·58 | 0·49 | 0·57 | 0·50 |
| Age |  |  |  |  |  |  |
| 60+ | 0·31 | 0·46 | 0·07 | 0·26 | 0·13 | 0·34 |
| 45-59 | 0·27 | 0·44 | 0·20 | 0·40 | 0·22 | 0·42 |
| 30-44 | 0·23 | 0·42 | 0·44 | 0·50 | 0·27 | 0·44 |
| 18-29 | 0·19 | 0·39 | 0·29 | 0·45 | 0·38 | 0·49 |
| Ethnicity |  |  |  |  |  |  |
| White | 0·88 | 0·33 | 0·85 | 0·36 | 0·80 | 0·40 |
| Ethnic minority groups | 0·12 | 0·33 | 0·15 | 0·36 | 0·20 | 0·40 |
| Education |  |  |  |  |  |  |
| Undergraduate degree or higher | 0·35 | 0·48 | 0·31 | 0·46 | 0·22 | 0·42 |
| A-levels or vocational | 0·33 | 0·47 | 0·40 | 0·49 | 0·44 | 0·50 |
| Up to GCSE | 0·32 | 0·47 | 0·29 | 0·45 | 0·33 | 0·47 |
| Income |  |  |  |  |  |  |
| >£90,000 | 0·07 | 0·26 | 0·05 | 0·21 | 0·08 | 0·27 |
| £60,000-89,999 | 0·13 | 0·34 | 0·05 | 0·22 | 0·06 | 0·24 |
| £30,000-59,999 | 0·33 | 0·47 | 0·45 | 0·50 | 0·20 | 0·40 |
| £16,000-29,999 | 0·28 | 0·45 | 0·27 | 0·44 | 0·37 | 0·48 |
| <£16,000 | 0·18 | 0·38 | 0·18 | 0·39 | 0·29 | 0·45 |
| Employment status |  |  |  |  |  |  |
| Not employed | 0·43 | 0·49 | 0·18 | 0·38 | 0·33 | 0·47 |
| Employed | 0·57 | 0·49 | 0·82 | 0·38 | 0·67 | 0·47 |
| Living arrangement |  |  |  |  |  |  |
| Alone | 0·20 | 0·40 | 0·18 | 0·39 | 0·20 | 0·40 |
| With others, not with children | 0·60 | 0·49 | 0·42 | 0·49 | 0·46 | 0·50 |
| With, including children | 0·20 | 0·40 | 0·40 | 0·49 | 0·34 | 0·47 |
| Area of dwelling |  |  |  |  |  |  |
| Urban | 0·79 | 0·41 | 0·84 | 0·37 | 0·81 | 0·40 |
| Rural | 0·21 | 0·41 | 0·16 | 0·37 | 0·19 | 0·40 |
| Smoking status |  |  |  |  |  |  |
| Former or non-smoker | 0·89 | 0·31 | 0·81 | 0·39 | 0·86 | 0·35 |
| Current smoker | 0·11 | 0·31 | 0·19 | 0·39 | 0·14 | 0·35 |
| Key worker status |  |  |  |  |  |  |
| Not a key worker | 0·79 | 0·41 | 0·72 | 0·45 | 0·69 | 0·46 |
| Key worker | 0·21 | 0·41 | 0·28 | 0·45 | 0·31 | 0·46 |
| Long-term physical health condition |  |  |  |  |  |  |
| No long-term physical health condition | 0·58 | 0·49 | 0·77 | 0·42 | 0·72 | 0·45 |
| Long-term physical health condition | 0·42 | 0·49 | 0·23 | 0·42 | 0·28 | 0·45 |
| Long-term mental health condition |  |  |  |  |  |  |
| No long-term mental health condition | 0·81 | 0·39 | 0·80 | 0·40 | 0·79 | 0·41 |
| Long-term mental health condition | 0·19 | 0·39 | 0·20 | 0·40 | 0·21 | 0·41 |
| Confidence in government to handle pandemic |  |  |  |  |  |  |
| Low confidence in government to handle pandemic | 0·36 | 0·48 | 0·24 | 0·43 | 0·34 | 0·47 |
| Low confidence in government to handle pandemic | 0·64 | 0·48 | 0·76 | 0·43 | 0·66 | 0·47 |
| Confidence in health system to handle pandemic |  |  |  |  |  |  |
| High confidence in health system to handle pandemic | 0·67 | 0·47 | 0·53 | 0·50 | 0·65 | 0·48 |
| Low confidence in health system to handle pandemic | 0·33 | 0·47 | 0·47 | 0·50 | 0·35 | 0·48 |
| Knowledge of COVID 19 |  |  |  |  |  |  |
| High knowledge of COVID-19 | 0·84 | 0·37 | 0·63 | 0·48 | 0·67 | 0·47 |
| Low knowledge of COVID-19 | 0·16 | 0·37 | 0·37 | 0·48 | 0·33 | 0·47 |
| Compliance across strict restriction periods |  |  |  |  |  |  |
| Consistently high compliance with COVID-19 guidelines | 0·67 | 0·47 | 0·45 | 0·50 | 0·35 | 0·48 |
| Consistently low compliance with COVID-19 guidelines | 0·33 | 0·47 | 0·55 | 0·50 | 0·65 | 0·48 |
| Have had COVID-19 |  |  |  |  |  |  |
| Have not had COVID-19 | 0·78 | 0·42 | 0·68 | 0·47 | 0·69 | 0·46 |
| Have had COVID-19, suspected by self or doctor | 0·10 | 0·31 | 0·14 | 0·35 | 0·12 | 0·32 |
| Have had COVID-19, confirmed by test | 0·12 | 0·32 | 0·18 | 0·38 | 0·19 | 0·39 |
| Stressed in the past week about catching/becoming ill from COVID-19 |  |  |  |  |  |  |
| Stressed | 0·33 | 0·47 | 0·22 | 0·42 | 0·18 | 0·38 |
| Not stressed | 0·67 | 0·47 | 0·78 | 0·42 | 0·82 | 0·38 |
| Initial COVID-19 vaccine intention |  |  |  |  |  |  |
| Initially willing to accept or had accepted a COVID-19 vaccine | 0·92 | 0·27 | 0·63 | 0·48 | 0·59 | 0·49 |
| Initially uncertain about accepting a COVID-19 vaccine | 0·05 | 0·23 | 0·24 | 0·43 | 0·23 | 0·42 |
| Initially unwilling to accept a COVID-19 vaccine | 0·02 | 0·15 | 0·13 | 0·33 | 0·19 | 0·39 |

Note. ‘Very likely’ to accept a COVID-19 booster vaccine was the reference group in the multinomial regression model. Ethnic minority groups refers to Black, Asian and minority ethnicity. GCSE refers to General Certificate of Secondary Education. Data were weighted to the proportions of gender, age, ethnicity, country, and education obtained from the Office for National Statistics.

**Table S5. Sensitivity analysis: Socio-demographic, COVID-19 related, and initial COVID-19 vaccine intent predictors of uncertainty and unwillingness to receive a COVID-19 booster vaccine using a multivariable multinomial regression and last recorded initial COVID-19 vaccine intent (weighted, *N* = 22,319)**

|  | **Uncertain** | | | **Unwilling** | | |
| --- | --- | --- | --- | --- | --- | --- |
|  | **RRR** | **95% CI** | | **RRR** | **95% CI** | |
| Gender (ref male) |  |  |  |  |  |  |
| Female | 1·12 | 0·77 | 1·62 | 1·06 | 0·68 | 1·65 |
| Age (ref 60+) |  |  |  |  |  |  |
| 45-59 | 1·43 | 0·90 | 2·25 | 1·33 | 0·77 | 2·28 |
| 30-44 | 3·09 | 1·82 | 5·25 | 1·61 | 0·81 | 3·21 |
| 18-29 | 3·20 | 1·76 | 5·83 | 3·24 | 1·46 | 7·23 |
| Ethnicity (ref white) |  |  |  |  |  |  |
| Ethnic minority groups | 0·80 | 0·42 | 1·50 | 1·13 | 0·53 | 2·42 |
| Education (ref undergraduate degree or higher) |  |  |  |  |  |  |
| A-levels or vocational | 1·39 | 0·87 | 2·20 | 1·69 | 0·98 | 2·92 |
| Up to GCSE | 1·57 | 1·00 | 2·45 | 2·03 | 1·14 | 3·59 |
| Income (ref >£90,000) |  |  |  |  |  |  |
| £60,000-89,999 | 0·49 | 0·20 | 1·23 | 0·34 | 0·12 | 0·99 |
| £30,000-59,999 | 2·54 | 1·10 | 5·87 | 0·69 | 0·29 | 1·66 |
| £16,000-29,999 | 2·27 | 0·89 | 5·79 | 1·41 | 0·53 | 3·78 |
| <£16,000 | 3·40 | 1·25 | 9·19 | 2·37 | 0·89 | 6·30 |
| Employment status (ref not employed) |  |  |  |  |  |  |
| Employed | 3·29 | 2·10 | 5·14 | 1·57 | 0·91 | 2·71 |
| Living arrangement (ref alone) |  |  |  |  |  |  |
| Living with others, not including children | 0·83 | 0·47 | 1·45 | 0·83 | 0·45 | 1·53 |
| Living with others, including children | 1·76 | 0·95 | 3·25 | 1·66 | 0·87 | 3·16 |
| Area of dwelling (ref urban) |  |  |  |  |  |  |
| Rural | 0·86 | 0·56 | 1·34 | 1·01 | 0·55 | 1·87 |
| Smoking status (ref former or non-smoker) |  |  |  |  |  |  |
| Current smoker | 1·65 | 1·02 | 2·68 | 1·22 | 0·63 | 2·35 |
| Key worker status (ref not a key worker) |  |  |  |  |  |  |
| Key worker | 0·74 | 0·49 | 1·13 | 1·20 | 0·67 | 2·15 |
| Long-term physical health condition (ref condition) |  |  |  |  |  |  |
| No long-term physical health condition | 1·41 | 0·98 | 2·05 | 1·09 | 0·71 | 1·68 |
| Long-term mental health condition (ref condition) |  |  |  |  |  |  |
| No long-term mental health condition | 1·34 | 0·87 | 2·06 | 1·52 | 0·83 | 2·79 |
| Confidence in government to handle pandemic (ref high) |  |  |  |  |  |  |
| Low confidence in government to handle pandemic | 1·19 | 0·81 | 1·76 | 0·85 | 0·51 | 1·42 |
| Confidence in health system to handle pandemic (ref high) |  |  |  |  |  |  |
| Low confidence in health system to handle pandemic | 1·32 | 0·91 | 1·93 | 0·83 | 0·53 | 1·30 |
| Knowledge of COVID 19 (ref high) |  |  |  |  |  |  |
| Low knowledge of COVID-19 | 1·81 | 1·22 | 2·68 | 1·11 | 0·63 | 1·95 |
| Compliance across strict restriction periods (ref high) |  |  |  |  |  |  |
| Consistently low compliance with COVID-19 guidelines | 1·58 | 1·09 | 2·31 | 2·82 | 1·87 | 4·25 |
| Have had COVID-19 (ref have not had COVID-19) |  |  |  |  |  |  |
| Have had COVID-19, suspected by self or doctor | 1·37 | 0·87 | 2·14 | 1·30 | 0·80 | 2·14 |
| Have had COVID-19, confirmed by test | 1·16 | 0·69 | 1·93 | 1·12 | 0·63 | 1·99 |
| Stressed in the past week about catching/becoming ill from COVID-19 (ref stressed) |  |  |  |  |  |  |
| Not stressed | 1·79 | 1·17 | 2·75 | 1·85 | 1·15 | 2·95 |
| Initial COVID-19 vaccine intention (ref willing) |  |  |  |  |  |  |
| Initially uncertain about accepting a COVID-19 vaccine | 4·41 | 2·08 | 9·35 | 10·86 | 5·18 | 22·78 |
| Initially unwilling to accept a COVID-19 vaccine | 6·47 | 2·46 | 17·02 | 10·67 | 3·98 | 28·59 |

Note. ‘Very likely’ to accept a COVID-19 booster vaccine was the reference group in the multinomial regression model. Ethnic minority groups refers to Black, Asian and minority ethnicity. GCSE refers to General Certificate of Secondary Education. Data were weighted to the proportions of gender, age, ethnicity, country, and education obtained from the Office for National Statistics.

**Table S6. Sensitivity analysis: Socio-demographic, COVID-19 related, and initial COVID-19 vaccine intent predictors of uncertainty and unwillingness to receive a COVID-19 booster vaccine using a multivariable multinomial regression using average compliance with government guidelines across the entirety of the pandemic (weighted, *N* = 22,139)**

|  | **Uncertain** | | | **Unwilling** | | |
| --- | --- | --- | --- | --- | --- | --- |
|  | **RRR** | **95% CI** | | **RRR** | **95% CI** | |
| Gender (ref male) |  |  |  |  |  |  |
| Female | 1·05 | 0·72 | 1·53 | 0·98 | 0·64 | 1·50 |
| Age (ref 60+) |  |  |  |  |  |  |
| 45-59 | 1·45 | 0·91 | 2·31 | 1·46 | 0·83 | 2·56 |
| 30-44 | 3·33 | 1·97 | 5·64 | 2·05 | 1·01 | 4·16 |
| 18-29 | 3·91 | 2·14 | 7·12 | 4·73 | 2·12 | 10·52 |
| Ethnicity (ref white) |  |  |  |  |  |  |
| Ethnic minority groups | 0·77 | 0·40 | 1·48 | 1·18 | 0·59 | 2·35 |
| Education (ref undergraduate degree or higher) |  |  |  |  |  |  |
| A-levels or vocational | 1·33 | 0·84 | 2·13 | 1·78 | 1·04 | 3·06 |
| Up to GCSE | 1·47 | 0·93 | 2·30 | 2·12 | 1·20 | 3·77 |
| Income (ref >£90,000) |  |  |  |  |  |  |
| £60,000-89,999 | 0·50 | 0·20 | 1·26 | 0·34 | 0·11 | 1·06 |
| £30,000-59,999 | 2·52 | 1·08 | 5·85 | 0·70 | 0·28 | 1·74 |
| £16,000-29,999 | 2·22 | 0·87 | 5·70 | 1·39 | 0·52 | 3·71 |
| <£16,000 | 3·07 | 1·13 | 8·29 | 2·09 | 0·78 | 5·56 |
| Employment status (ref not employed) |  |  |  |  |  |  |
| Employed | 3·15 | 2·00 | 4·94 | 1·43 | 0·84 | 2·46 |
| Living arrangement (ref alone) |  |  |  |  |  |  |
| Living with others, not including children | 0·73 | 0·42 | 1·28 | 0·72 | 0·39 | 1·33 |
| Living with others, including children | 1·67 | 0·89 | 3·12 | 1·61 | 0·85 | 3·08 |
| Area of dwelling (ref urban) |  |  |  |  |  |  |
| Rural | 0·87 | 0·55 | 1·36 | 1·01 | 0·56 | 1·81 |
| Smoking status (ref former or non-smoker) |  |  |  |  |  |  |
| Current smoker | 1·79 | 1·11 | 2·91 | 1·27 | 0·66 | 2·44 |
| Key worker status (ref not a key worker) |  |  |  |  |  |  |
| Key worker | 0·74 | 0·48 | 1·14 | 1·21 | 0·67 | 2·18 |
| Long-term physical health condition (ref condition) |  |  |  |  |  |  |
| No long-term physical health condition | 1·40 | 0·96 | 2·04 | 1·18 | 0·77 | 1·79 |
| Long-term mental health condition (ref condition) |  |  |  |  |  |  |
| No long-term mental health condition | 1·37 | 0·88 | 2·13 | 1·54 | 0·86 | 2·75 |
| Confidence in government to handle pandemic (ref high) |  |  |  |  |  |  |
| Low confidence in government to handle pandemic | 1·19 | 0·81 | 1·75 | 0·87 | 0·53 | 1·44 |
| Confidence in health system to handle pandemic (ref high) |  |  |  |  |  |  |
| Low confidence in health system to handle pandemic | 1·32 | 0·90 | 1·94 | 0·85 | 0·54 | 1·34 |
| Knowledge of COVID 19 (ref high) |  |  |  |  |  |  |
| Low knowledge of COVID-19 | 1·78 | 1·19 | 2·67 | 1·19 | 0·70 | 2·05 |
| Compliance across strict restriction periods (ref high) |  |  |  |  |  |  |
| Consistently low compliance with COVID-19 guidelines | 1·51 | 1·04 | 2·19 | 2·45 | 1·65 | 3·65 |
| Have had COVID-19 (ref have not had COVID-19) |  |  |  |  |  |  |
| Have had COVID-19, suspected by self or doctor | 1·38 | 0·89 | 2·15 | 1·24 | 0·76 | 2·03 |
| Have had COVID-19, confirmed by test | 1·11 | 0·66 | 1·88 | 1·06 | 0·59 | 1·91 |
| Stressed in the past week about catching/becoming ill from COVID-19 (ref stressed) |  |  |  |  |  |  |
| Not stressed | 1·80 | 1·19 | 2·72 | 1·91 | 1·19 | 3·07 |
| Initial COVID-19 vaccine intention (ref willing) |  |  |  |  |  |  |
| Initially uncertain about accepting a COVID-19 vaccine | 4·92 | 2·98 | 8·11 | 5·29 | 3·07 | 9·09 |
| Initially unwilling to accept a COVID-19 vaccine | 6·40 | 3·94 | 10·41 | 11·29 | 6·79 | 18·78 |

Note. ‘Very likely’ to accept a COVID-19 booster vaccine was the reference group in the multinomial regression model. Ethnic minority groups refers to Black, Asian and minority ethnicity. GCSE refers to General Certificate of Secondary Education. Data were weighted to the proportions of gender, age, ethnicity, country, and education obtained from the Office for National Statistics.

**Table S7. Sensitivity analysis: Socio-demographic, COVID-19 related, and initial COVID-19 vaccine intent predictors of uncertainty and unwillingness to receive a COVID-19 booster vaccine using a multivariable multinomial regression including only participants who had had exactly two doses of a COVID-19 vaccine at follow-up (weighted, *N* = 12,297)**

|  | **Uncertain** | | | **Unwilling** | | |
| --- | --- | --- | --- | --- | --- | --- |
|  | **RRR** | **95% CI** | | **RRR** | **95% CI** | |
| Gender (ref male) |  |  |  |  |  |  |
| Female | 1·12 | 0·76 | 1·63 | 1·04 | 0·67 | 1·60 |
| Age (ref 60+) |  |  |  |  |  |  |
| 45-59 | 0·82 | 0·50 | 1·34 | 1·06 | 0·59 | 1·91 |
| 30-44 | 1·69 | 0·98 | 2·91 | 1·29 | 0·62 | 2·66 |
| 18-29 | 1·85 | 0·97 | 3·50 | 2·69 | 1·15 | 6·30 |
| Ethnicity (ref white) |  |  |  |  |  |  |
| Ethnic minority groups | 0·85 | 0·44 | 1·64 | 1·15 | 0·57 | 2·31 |
| Education (ref undergraduate degree or higher) |  |  |  |  |  |  |
| A-levels or vocational | 1·24 | 0·77 | 1·99 | 1·70 | 0·98 | 2·95 |
| Up to GCSE | 1·37 | 0·86 | 2·20 | 1·83 | 1·00 | 3·34 |
| Income (ref >£90,000) |  |  |  |  |  |  |
| £60,000-89,999 | 0·55 | 0·23 | 1·35 | 0·34 | 0·11 | 1·08 |
| £30,000-59,999 | 2·49 | 1·08 | 5·75 | 0·71 | 0·28 | 1·79 |
| £16,000-29,999 | 2·20 | 0·86 | 5·67 | 1·50 | 0·55 | 4·06 |
| <£16,000 | 2·91 | 1·06 | 7·97 | 1·95 | 0·71 | 5·33 |
| Employment status (ref not employed) |  |  |  |  |  |  |
| Employed | 3·12 | 1·92 | 5·06 | 1·49 | 0·85 | 2·62 |
| Living arrangement (ref alone) |  |  |  |  |  |  |
| Living with others, not including children | 0·77 | 0·43 | 1·38 | 0·83 | 0·41 | 1·68 |
| Living with others, including children | 1·69 | 0·89 | 3·23 | 1·77 | 0·86 | 3·61 |
| Area of dwelling (ref urban) |  |  |  |  |  |  |
| Rural | 0·85 | 0·53 | 1·34 | 1·03 | 0·54 | 1·94 |
| Smoking status (ref former or non-smoker) |  |  |  |  |  |  |
| Current smoker | 1·95 | 1·18 | 3·22 | 1·43 | 0·73 | 2·79 |
| Key worker status (ref not a key worker) |  |  |  |  |  |  |
| Key worker | 0·91 | 0·58 | 1·41 | 1·45 | 0·80 | 2·63 |
| Long-term physical health condition (ref condition) |  |  |  |  |  |  |
| No long-term physical health condition | 1·07 | 0·71 | 1·60 | 0·90 | 0·57 | 1·41 |
| Long-term mental health condition (ref condition) |  |  |  |  |  |  |
| No long-term mental health condition | 1·39 | 0·88 | 2·21 | 1·49 | 0·81 | 2·74 |
| Confidence in government to handle pandemic (ref high) |  |  |  |  |  |  |
| Low confidence in government to handle pandemic | 1·13 | 0·76 | 1·69 | 0·88 | 0·52 | 1·50 |
| Confidence in health system to handle pandemic (ref high) |  |  |  |  |  |  |
| Low confidence in health system to handle pandemic | 1·33 | 0·90 | 1·97 | 0·85 | 0·53 | 1·37 |
| Knowledge of COVID 19 (ref high) |  |  |  |  |  |  |
| Low knowledge of COVID-19 | 1·73 | 1·15 | 2·61 | 1·14 | 0·65 | 2·01 |
| Compliance across strict restriction periods (ref high) |  |  |  |  |  |  |
| Consistently low compliance with COVID-19 guidelines | 1·50 | 1·03 | 2·20 | 2·43 | 1·61 | 3·66 |
| Have had COVID-19 (ref have not had COVID-19) |  |  |  |  |  |  |
| Have had COVID-19, suspected by self or doctor | 1·23 | 0·78 | 1·94 | 1·12 | 0·67 | 1·87 |
| Have had COVID-19, confirmed by test | 1·07 | 0·63 | 1·83 | 1·16 | 0·63 | 2·12 |
| Stressed in the past week about catching/becoming ill from COVID-19 (ref stressed) |  |  |  |  |  |  |
| Not stressed | 1·90 | 1·24 | 2·91 | 1·83 | 1·11 | 3·00 |
| Initial COVID-19 vaccine intention (ref willing) |  |  |  |  |  |  |
| Initially uncertain about accepting a COVID-19 vaccine | 4·62 | 2·77 | 7·70 | 5·31 | 3·05 | 9·25 |
| Initially unwilling to accept a COVID-19 vaccine | 5·36 | 3·25 | 8·84 | 9·15 | 5·29 | 15·84 |

Note. ‘Very likely’ to accept a COVID-19 booster vaccine was the reference group in the multinomial regression model. Ethnic minority groups refers to Black, Asian and minority ethnicity. GCSE refers to General Certificate of Secondary Education. Data were weighted to the proportions of gender, age, ethnicity, country, and education obtained from the Office for National Statistics.
