# Supplementary material for: Predictors of uncertainty and unwillingness to receive the COVID-19 booster vaccine: An observational study of 22,139 fully vaccinated adults in the UK
Source: Lancet Reg Health Eur. 2022 Feb 3;14:100317. doi: 10.1016/j.lanepe.2022.100317 (PMC8811487; doi:10.1016/j.lanepe.2022.100317)
Supplement: Supplementary file 2 [file mmc2.docx]

**Supplementary Material**

Table S1. Comparison of excluded and included participants, unweighted

Table S2. Wording of study developed items

Table S3. Dates of strict restrictions in England

Table S4. Comparison of sample participants by COVID-19 booster vaccine intention (weighted, *N* = 22,139)

Table S5. Sensitivity analysis: Socio-demographic, COVID-19 related, and initial COVID-19 vaccine intent predictors of uncertainty and unwillingness to receive a COVID-19 booster vaccine using a multivariable multinomial regression and last recorded initial COVID-19 vaccine intent **(**weighted, *N* = 22,319)

Table S6. Sensitivity analysis: Socio-demographic, COVID-19 related, and initial COVID-19 vaccine intent predictors of uncertainty and unwillingness to receive a COVID-19 booster vaccine using a multivariable multinomial regression using average compliance with government guidelines across the entirety of the pandemic (weighted, *N* = 22,139)

Table S7. Sensitivity analysis: Socio-demographic, COVID-19 related, and initial COVID-19 vaccine intent predictors of uncertainty and unwillingness to receive a COVID-19 booster vaccine using a multivariable multinomial regression including only participants who had had exactly two doses of a COVID-19 vaccine at follow-up (weighted, *N* = 12,297)
